# Exploring the Role of the Processing Body in Plant Abiotic Stress Response

**DOI:** 10.3390/cimb46090585

**Published:** 2024-09-04

**Authors:** Zhehao Huang, Zhi Xu, Xiuqing Liu, Gangmin Chen, Chensi Hu, Menglu Chen, Yun Liu

**Affiliations:** Guangdong Provincial Key Laboratory for Plant Epigenetics, College of Life Sciences and Oceanography, Shenzhen University, Shenzhen 518060, China

**Keywords:** processing body, membrane-less organelle, protein composition, assembly of P-Body, abiotic stress, ethylene signaling, stress resistance, post-transcriptional regulation

## Abstract

The processing body (P-Body) is a membrane-less organelle with stress-resistant functions. Under stress conditions, cells preferentially translate mRNA that favors the stress response, resulting in a large number of transcripts unfavorable to the stress response in the cytoplasm. These non-translating mRNAs aggregate with specific proteins to form P-Bodies, where they are either stored or degraded. The protein composition of P-Bodies varies depending on cell type, developmental stage, and external environmental conditions. This review primarily elucidates the protein composition in plants and the assembly of P-Bodies, and focuses on the mechanisms by which various proteins within the P-Bodies of plants regulate mRNA decapping, degradation, translational repression, and storage at the post-transcriptional level in response to ethylene signaling and abiotic stresses such as drought, high salinity, or extreme temperatures. This overview provides insights into the role of the P-Body in plant abiotic stress responses.

## 1. Introduction

Various complex biochemical reactions within cells take place in separate cellular compartments. Apart from membrane-bound organelles, there also exist membrane-less organelles (MLOs) formed through liquid–liquid phase separation (LLPS) involving proteins and nucleic acids [1,2]. These MLOs compartmentalize within the cell to provide stable and non-interfering internal environments for specific biochemical reactions, such as the nucleolus in the cell nucleus, Cajal bodies, stress granule (SG), and processing body (P-Body). Parker et al. first discovered in yeast that mRNA can be decapped and 5′–3′ degraded in some discrete cytoplasmic foci and called these cytoplasmic foci P-Bodies [3,4,5]. Subsequent research found that P-Bodies are formed from aggregation among proteins and mRNA molecules driven by LLPS under specific conditions, such as pH, salt concentration, and temperature [6,7]. The P-Body is widely distributed in different cell types.

The P-Body is primarily involved in mRNA decapping, translational repression, degradation, and storage at the post-transcriptional level [8]. The mRNA degradation mediated by P-Bodies includes the AU-rich element-mediated mRNA decay (AMD) pathway, the 5′–3′ degradation pathway, and the nonsense-mediated mRNA decay (NMD) [9,10]. mRNA degradation not only regulates the expression levels of normal proteins but also prevents the synthesis of abnormally folded proteins. Notably, not all mRNAs in P-Bodies are degraded; several mRNAs can be stored in the P-Body under abiotic stresses and re-enter the polysomes when conditions become favorable, restoring translation. For instance, mRNA levels in P-Bodies were elevated in glucose deficiency and decreased after glucose supplementation by a shift in the cellular polysome count from a decrease to an increase [11].

The P-Body has been reported to play a crucial role in various cell types. The results in mammals indicate that the P-Body is involved in defense against viral infections and the regulation of oocyte maturation [12,13,14]. In plants, the P-Body also plays a significant role in regulating cellular physiological activities. During seed germination, the unnecessary mRNAs for seedling growth were degraded in the P-Body; then, the ribosomal translation sites were free for synthesizing essential proteins to sustain plant normal growth and development [15]. Additionally, P-Bodies are indispensable in plant responses to stress. Under heat, salt, and pathogen stress conditions, the size and number of P-Bodies increased, and the protein composition of the P-Bodies degrades mRNAs unfavorable to the stress response [16,17,18]. The protein composition of the P-Body varies in different cell types, growth stages, and external environmental stresses. The review primarily elucidates the protein composition of P-Bodies in plants, the protein structures that drive the formation of P-Bodies, and the mechanisms of P-Body assembly. Furthermore, we focus on how P-Bodies regulate mRNA decapping, degradation, translational repression, and storage in response to various abiotic stresses and the involvement in hormone signaling pathways. The review summarizes the dynamic changes and response mechanisms of P-Bodies in plants under abiotic stresses.

## 2. Components of P-Body

The P-Body is composed of mRNA and proteins. Although there have been many studies on the components of the P-Body, to date, the composition of the P-Body is not completely clear, as many components can move in and out of the P-Body dynamically. Many components of the P-Body shuttle in and out dynamically, leading to variability in composition across cell types, growth stages, and environmental conditions. The proteins in the P-Body could be divided to conserved and dynamic proteins [19]. Due to the conservation of P-Body protein components in eukaryotes, many proteins in the P-Body of *Arabidopsis thaliana* such as Decapping Protein 1 (DCP1), Decapping Protein 2 (DCP2), and ARGONAUTE (AGO), are found to be homologous to those in yeast or mammalian cells [20,21].

### 2.1. The Conserved Protein Components of P-Bodies in Plants

The conserved proteins in the P-Bodies of plants include exoribonuclease XRN4 (homologous to the exoribonuclease XRN1 found in mammals and yeast), decapping enzyme DCP2, and the uncapping activator VARICOSE (VCS), Like Sm 1–7 (LSM1) complex, DCP1, Decapping Protein 5 (DCP5), etc. [22]. They are associated with mRNA decapping, degradation, and protein translation repression [10,22]. In decapping-deficient mutants of *DCP1, DCP2*, and *DCP5,* mRNA encoding seed storage protein (SSP) cannot be decapped and degraded in the P-Body during seed germination. These mRNAs re-associate with polysomes for translation, thereby preventing the translation of newly synthesized mRNAs, which produce proteins necessary for seedling growth [23]. The expression of proteins required for seedling growth is inhibited leading to growth inhibition or even the death of plants at the seedling stage [15]. This indicates that the mRNA decapping and degradation mechanism mediated by the P-Body-conserved proteins plays a necessary role in the transition from the seed to the seedling growth stage in plants and is required for normal plant growth.

### 2.2. The Dynamic Protein Components of P-Body in Plants

The composition of the P-Body varies in different types or developmental stages of organs. *Arabidopsis* WD40-REPEAT 5a (WDR5a) localizes in the P-Body of the juvenile leaf cell during cytokinesis and early differentiation, while in root cells, it does not localize in the P-Body [24]. Furthermore, environmental stresses can also affect the components of the P-Body. Under drought stress conditions, the tandem CCCH zinc finger (TZF) protein in *Oryza sativa* relocates from the nucleus to the P-Body and stress granules (SGs). This movement enhances drought tolerance through regulating the expression of drought-related genes at both the transcriptional and post-transcriptional levels [25,26,27]. Similarly, under heat stress, the ALBA protein in *Arabidopsis* can specifically recruit the mRNA of heat stress transcription factors (HSFs) to the P-Body and protect them from degradation. HSFs are crucial in regulating the expression of heat-responsive genes, thereby enhancing plant thermotolerance [28]. Consequently, the complex regulatory mechanisms of the P-Body in plants are mediated by the dynamic protein composition of P-Body changes with cell type, growth stage, and external environment. The dynamic protein of the P-Body further strengthens the resistance of different cells of plants to different stresses.

## 3. Assembly of P-Body

### 3.1. Liquid–Liquid Phase Separation (LLPS) Drives P-Body Assembly

Most of the proteins in membrane-less organelles (MLOs) are intrinsically disordered proteins or contain intrinsically disordered regions (IDRs) [29]. Some of these IDRs contain binding sites that promote multivalent interactions or a large number of low hydrophobic sequences that have the ability to drive proteins to undergo liquid–liquid phase separation and thus aggregate into MLOs [30,31]. As a type of MLO, the formation of the P-Body is also related to disordered protein structure. Currently, numerous studies focus on the protein components of the P-Body, utilizing bioinformatics predictions and nuclear magnetic resonance (NMR) experiments to analyze its protein structure. Table 1 lists the proteins of the P-Body that have been shown to have disordered regions. The intrinsically disordered structure of these proteins contributes to the proper localization of the proteins and participates in the assembly of the P-Body. PONDR http://pondr.com/ (accessed on 28 August 2024) predicts that the serine-rich C-terminal region of the Nst1 protein is disordered in yeast. Compared to the Nst1 protein, the *Nst1* mutants that lack the serine-rich region have inhibited the ability to form P-Bodies [32]. Similarly, in *Arabidopsis*, the SUPPRESSOR WITH MORPHOGENETIC EFFECTS ON GENITALIA 7 (SMG7) protein, which lacks a disordered C-terminus, was unable to correctly localize THREE-DIVISION MUTANT 1 (TDM1) to the P-Body upon interaction with TDM1 [33]. All of these results indicate that the intrinsically disordered structure of the proteins is essential for the formation of the P-Body.

In addition to intrinsically disordered regions (IDRs), proteins in the P-Body typically contain low-complexity sequences, RNA-binding activity, and prion-like domains, which promote LLPS and are crucial in the assembly and maintenance of the P-Body [19]. The RNA-binding proteins aggregate with mRNA to initially form mRNP. To form a larger quantity and size of P-Body, these mRNPs further interacted through domains such as Lsm4 Q/N domain, Edc3 Yjef-N domain, or Pat1 N Terminal Domain [19,44]. This complex interaction eventually forms visible particles in the cytoplasm. Yeast strains lacking either *Edc3* or the (C)-terminal Q/N-rich domain of the *Lsm4* can still form P-Bodies through another protein driving assembly, but the P-Body in the *Edc3Δ Lsm4ΔC* double deletion strain is drastically reduced [44]. In addition, Decker et al. replaced the C-terminal structural domain of Lsm4 with a prion-like structural domain of Rnq1, and found that the number and volume of the P-Bodies in the *Edc3Δ Lsm4ΔC* double deletion strain was restored to normal levels, proving that the C terminus of Lsm4 is rich in Q/N, the domain of which is a prion-like domain [45]. The above results also suggest that phase separation driven by domains containing low-complexity sequences, RNA-binding activity, or prion-like structural domains is critical in the assembly of the P-Body.

### 3.2. P-Body Specific Sequence and Mechanism of Assembly

During the process of protein–mRNA interaction, there are certain proteins that serve as scaffolds or recruit mRNA and other protein components which are essential for phase separation in P-Body [46]. For example, to form the decapping complexes of P-Bodies, the N- or C-terminal region of the VCS protein could interact with DCP1 and DCP2, respectively, in the cytoplasm of the *Arabidopsis* late embryonic development stage [15]. Moreover, DCP5 can inhibit mRNA translation and aggregate with it to form mRNP, then DCP1 and DCP2 are recruited by the C-terminal region of the DCP5 protein containing RGG and FDF domains [23]. The results of immunoprecipitation showed that the DCP5 could interact with DCP1 and DCP2 but not the VCS protein [23]. This suggests that the assembly of P-Bodies is not merely a simple aggregation of proteins and mRNAs, but rather follows a specific sequence and mechanism of assembly.

### 3.3. RNA Decay Is the Main Reason for the Formation of P-Body

In cells, the P-Body mainly performs the degradation and storage functions of mRNA and plays a crucial role in the silencing and decay of mRNA [47]. Therefore, the formation and disassembly of the P-Body are directly related to the mRNA in the cytoplasm. When cells are in a specific growth stage or under stress conditions, a large amount of mRNA often appears in the cytoplasm. These mRNAs will participate in the formation of P-Bodies and promote the growth of the number and volume of P-Bodies. For example, the P-Bodies begin to increase in number as the cell approaches the S phase of cytokinesis until the end of interphase, and then breaks down during mitosis [48]. This may be because during the S phase of cell division, the transcription rate is very high, necessitating the involvement of the P-Body in mRNA degradation to regulate the quantity of mRNA and precisely control protein synthesis [48,49]. However, during cell division, the P-Body disassembles to ensure there is sufficient mRNA to rapidly translate and synthesize proteins to support cell growth once cell division is complete [48].

Under stress conditions, in order to preferentially translate more new stress-related mRNAs, cells will inhibit the translation of some mRNAs that are not favorable to the stress response or are not suitable for mass translation under stress conditions. These mRNAs are involved in the formation of the P-Body and are then degraded or stored in the P-Body. When the cell’s growth environment returns to normal conditions, the P-Body in the cytoplasm breaks down, and the mRNAs stored in the P-Body return to the ribosomes to be translated again. The cell’s mRNAs that have been repressed in the cytoplasm are then degraded and stored in the P-Body, and the mRNAs that have been repressed are then returned to the ribosomes for translation [4]. In addition, the P-Body can also resist RNA virus infection of cells through the mRNA degradation pathway. At the same time, the P-Body is also the target of RNA virus attacks. Kleer et al. used three RNA viruses, SARS-CoV-2, OC43, and 229E, to infect human umbilical vein endothelial cells (HUVECs) for 24 h and found that the amount of P-Body in the cytoplasm was significantly reduced [50]. It is worth noting that while the P-Body is depolymerized, inflammatory cytokine transcripts will also return to cytoplasmic ribosomes for translation, thereby alerting and activating the immune system to enhance resistance to viruses [50]. The aforementioned results indicate that the formation of P-Bodies is related to RNA decay. Additionally, studies have shown that the aggregation of P-Body components is associated with the microtubule network, an essential structure of the cytoskeleton. Hurst et al. discovered that disrupting the microtubules of yeast cells with 15 μg/mL benomyl for 60 min led to an increase in the levels of P-Body proteins such as Dhh1p, Dcp2p, and Xrn1, subsequently leading to the aggregation and formation of P-Bodies [50,51,52]. Notably, the disruption of cellular microtubules does not result in the inhibition of mRNA translation [51]. At present, it is not clear why damaging cellular microtubules leads to P-Body aggregation, but it may be related to the fact that microtubules restrict the movement of P-Bodies [49].

## 4. P-Body and Plant Stress Responses

Under abiotic stress, the number and size of the P-Bodies increased significantly in plant cells to degrade and store mRNAs that are unfavorable for stress responses [17]. In addition to conserved proteins such as DCP1, DCP2, and DCP5, the proteins involved in the formation of the P-Bodies also include some dynamic proteins that only aggregate into P-Bodies under specific stresses such as drought, salinity, heat, and cold. Currently, numerous studies have explored the functions of these dynamic proteins by subjecting plants such as *Arabidopsis thaliana*, *Oryza sativa*, and *Nicotiana tabacum* to specific abiotic stresses. For instance, *Os*CAF1B protein was induced to express under cold stress condition in *Oryza sativa* seedlings. *Os*CAF1B has been shown to have deadenylase activity, which may promote the degradation of mRNA that is not conducive to cold stress by participating in the deadenylation of mRNA in P-Bodies [53,54].

### 4.1. Response of P-Body to Drought and Salt Stress

In plants, the P-Body responds to drought or salt stress through modulating 5′→3′ mRNA degradation. Under drought or salt stress, transcripts undergo a deadenylation reaction catalyzed by poly(A)-specific ribonuclease (PARN) in *Arabidopsis* [10]. In P-Bodies, the deadenylated transcripts are decapped by the decapping complex consisting of DCP1, DCP2, DCP5, VCS, etc. [10,22,23]. When the *Arabidopsis* seed was dehydrated for 15 min, MPK6 is activated and phosphorylates the Ser237 of the DCP1 protein. Phospho-DCP1 protein is preferentially associated with the RGG domain of DCP5 protein and promotes the interaction of the FDF domain of DCP5 with DCP2 protein to form the P-Body. And then the mRNA decapping was facilitated in the P-Body [23,55]. Moreover, the VCS protein acts as a scaffold protein to facilitate the aggregation of the P-Body. This occurs by binding the components of the P-Body proteins together, such as interacting with the DCP1 protein through the N-terminal region and with the DCP2 protein through the C-terminal region in *Arabidopsis* [10,15]. The decapped mRNA produced by the decapping complex is subsequently degraded by 5′→3′ exoribonuclease *At*XRN4 [10].

Similarly, the subclass I protein kinases SnRK2 proteins could phosphorylate VCS protein in the early stages under osmotic stress conditions. Phosphorylated VCS not only participates in 5′–3′ mRNA degradation but also promotes the accumulation of transcripts that respond to drought stress [15,56]. After 5 h of dehydration treatment, compared to the wild-type, the expression levels of 376 genes increased significantly in mutants lacking SnRK2 protein, while 519 genes decreased significantly [56]. Most of these up-/downregulated genes were genes whose expression was suppressed by drought and genes that favored drought resistance, respectively [56]. The results suggest that protein kinases modify the conformation of conserved P-Body proteins through phosphorylation, promoting their interaction and assembly into complexes with mRNA degradation functions, known as P-Body.

Apart from the complex composed of conserved proteins, CCCH-zinc finger proteins, which are dynamic proteins of the P-Body, also modulate plant growth and stress response by regulating the expression of drought-related genes at both transcriptional and post-transcriptional levels [57]. Under drought or salt conditions, members of the zinc finger protein family-*Os*TZF7, *Os*C3H10, *Os*TZF1, and *At*TZF1 can traffic between the nucleus and P-Body/stress granules [25,26,27,57]. The overexpression of *OsTZF7*, *OsC3H10,* or *OsTZF1* in *Oryza sativa* and *AtTZF1* in *Arabidopsis* results in significantly lower rates of water loss compared to wild-type plants after drought treatment, while the RNAi-mediated knockdown of these genes in *Oryza sativa* or *Arabidopsis* leads to slightly higher rates of water loss [25,26,27,58]. RNA-seq and DNA microarray analyses of OE and WT plants showed that transcripts of genes associated with dehydration response, such as metallothionein, ferritin, and dehydrins are significantly upregulated in OE lines [25,26,27,58].

*Os*TZF7, *Os*TZF1, and other TZF proteins have also been reported to be involved in salt stress response [25,27]. When the wild-type, *OsTZF1*-OX, and *OsTZF1*-RNAi *Oryza sativa* seedlings were subjected to 250 mM NaCl for 3 days, the survival ratios of *OsTZF1*-OX and control plants (WT) were over 65% and 33%, respectively, and the survival ratios of *OsTZF1*-OX was twice of that of the wild-type [27]. In contrast, 19% to 21% of the *OsTZF1*-RNAi plants survived, which is lower than that of the control lines [27]. In addition, DNA microarray analysis revealed that the transcripts of salt stress tolerance genes are upregulated significantly in *Os*TZF1-OX transgenic seedlings, such as RD22, YSL6, allene oxide synthase, etc. [27].

The above results indicate that CCCH-zinc finger proteins respond to abiotic stress by dynamically trafficking between the nucleus and P-Body/stress granules to regulate the expression of related stress-responsive genes at the transcriptional and post-transcriptional levels.

### 4.2. Response of P-Body to Cold or Heat Stress

In plants, P-Bodies are more sensitive to temperature stress, particularly heat stress. The number and volume of P-Bodies increases significantly under heat treatment [17]. Under normal growth conditions, *Arabidopsis* DCP1 and DCP2 are dispersed in the cytoplasm, but when exposed to 40 °C heat treatment, they aggregate into P-Body-like granules within the cytoplasm. When the heat treatment was removed, the P-Bodies disassembled gradually and their components diffused evenly in the cytoplasm [59]. As in drought stress, under temperature stress, the conserved proteins of the P-Body aggregate into a complex with 5′→3′ mRNA degradation function. During heat treatment, the 5′-m7Gpp protective structures of mRNAs that are not beneficial for heat adaptation are excised by the DCP1/2 complex [59]. The decapped mRNA is subsequently degraded by 5′→3′ exoribonuclease XRN4 [60].

It is worth noting that the content of the Decapping Protein (DCP) family in the *Arabidopsis* P-Body changes with the different temperature treatments. During heat treatment, the content of DCP2 is higher than that of DCP1. While under cold conditions, the content of DCP1 is higher than that of DCP2 [59]. It implies a differential regulation of mRNA decapping processes in the P-Body depending on the type of temperature stress. Specifically, DCP2 may be more actively involved in mRNA degradation under heat stress, whereas DCP1 may play a more prominent role in mRNA decapping and degradation under cold stress conditions. Brengues et al. found that Dcp2p, Dhh1p, PGK1-U1A, and MFA2P-U1A mRNAs exit P-Bodies for translation in yeast when recovered from glucose deprivation to normal physiological conditions, proving that the P-Body has the function of storing mRNA [11]. Therefore, besides mRNA degradation, changes in DCP1 and DCP2 content may mean that they have the function of storing mRNA [59]. This facilitates the rapid translation of mRNAs required for plant growth to adapt to normal environments.

In addition to mRNAs that are not beneficial for heat or cold adaptation, some aberrant or truncated mRNAs can also be degraded in the P-Body. Cold resistance could be enhanced in *Arabidopsis* by cold acclimation (CA). During CA, the transcripts induced by cold are translated into functional proteins which contributed to increasing the low-temperature tolerance. However, during the post-transcriptional regulation of mRNA, the occurrence rate of premature termination codons (PTCs) increased due to alternative splicing, generating aberrant mRNAs or truncated mRNAs. These mRNAs will be eventually degraded in the P-Body [61].

Besides P-Bodies, plants can form other membrane-less organelles (MLOs), such as stress granules (SGs) under heat stress [62,63]. When *Arabidopsis* was treated at 35 °C heat conditions for 70 s, SGs were fused with P-Bodies and the protein and mRNA exchanged frequently between them. The thermotolerance of plants is enhanced through both mRNA degradation and storage mechanisms [63]. Tudor Staphylococcal Nuclease (TSN) proteins are located in P-Body and SGs and are essential for the assembly of SGs and P-Body under heat-stress conditions [64]. Additionally, it can promote the decapping of mRNAs which are not conducive to heat stress tolerance [63,64]. In *Arabidopsis*, the acetylation lowers the binding affinity (ALBA) proteins ALBA4, ALBA5, and ALBA6 located in P-Bodies and SGs and directly binds them to the mRNA of heat stress transcription factors (HSFs) under heat stress, recruiting them to SGs and P-Bodies to protect the mRNA from being degraded [28]. The TZF family protein *Hu*TZF3 is located in P-Body and SGs and plays regulatory roles at the post-transcriptional level, then reduces the levels of reactive oxygen species (ROS) generated in response to salt or heat stress to enhance tolerance in *Hylocereus polyrhizus* [65]. P-Body and SGs synergistically regulate mRNA degradation and storage by exchanging components confirming the dynamic nature of P-Body.

### 4.3. P-Body Involving in Ethylene Signaling Pathway

Ethylene is a major signaling molecule in plants responding to biotic and abiotic stresses [66]. The key components of the P-Body, such as EIN5 and PAB2/4/8, contribute to the accumulation of critical transcription factors EIN3/EIL1 in the ethylene response pathway [66,67]. The overexpression of *EBF1* 3′ UTR region (*1U*) in *Arabidopsis* promotes the translation of *EBF1/2* mRNAs and reduces the sensitivity of the plant responding to ethylene [66]. With ethylene present, the CEND region of EIN2 binds to *1U*-containing mRNA and directs it to P-Body to interact with EIN5 and PAB2/4/8 to inhibit its translation. Consequently, the translation of *EBF1/2* mRNAs was inhibited, and the accumulation of EIN3/EIL1, which is responsive to ethylene, was promoted [66].

Additionally, in *Oryza sativa*, the MHZ9 protein is involved in the ethylene signaling pathway positively at the translation level [68]. In the presence of ethylene, MHZ9 together with *Os*EIN2-C is co-localized with the P-Body proteins *Os*DCP2 and *Os*EIN5. The binding of the N-terminal domain of MHZ9 to the 3′UTR region of *OsEBF1/2* mRNAs inhibits its translation, thereby promoting the expressions of the *OsEIL1* to activate the downstream ethylene signaling pathway [68]. Furthermore, the knockout mutants of genes encoding P-Body components (*EIN5*, *UPF1*, or *PAB2/8*) exhibit decreased sensitivity to ethylene. This insensitivity is especially pronounced in the *ein5 upf1 pab2 pab8* quadruple mutant. All these results indicate that P-Bodies play a critical role in the ethylene signaling pathway. Ethylene quickly shuts down *EBF1/2* gene expression through the mechanism of translation inhibition in the P-Body to regulate the stress responses [66].

In addition to participating in the ethylene signaling pathway, P-Bodies can also enhance stress resistance and regulate plant growth by interacting with stress-responsive proteins in the hormonal pathways or by promoting the expression of genes associated with other hormonal pathways. For example, in *Brassica napus*, BnRH6 in the P-Body upregulates the expression of ABA signaling pathway genes, including *CYP707A1*, *MAPKKK18*, *NCED9*, *AHG1*, etc., under salt stress [69]. In the P-Body, AtTZF4/5/6 could interact with Responsive to Dehydration 21A (RD21A) and the Mediator of ABA-Regulated Dormancy 1 (MARD1) to exert post-transcriptional level regulatory function in plants’ growth and responses to stress [70].

## 5. Conclusions and Perspectives

The P-Body responds to abiotic stresses in plant cells such as drought, salt, and temperature stress by regulating mRNA uncapping, degradation, translational repression, and storage at the post-transcriptional level. It also improves plant stress resistance by being involved in ethylene signaling pathways [8]. The formation of the P-Body is related to mRNA decay. Under stress conditions, plant cells prioritize the translation of mRNAs that favor the stress response by inhibiting the translation of transcripts that are less favorable [4]. These non-translating mRNAs form P-Bodies through liquid–liquid phase separation (LLPS) with proteins containing intrinsically disordered regions (IDRs), low-complexity sequences, RNA-binding activities, or prion-like domains, and are either stored within them or degraded [19,31]. In addition to mRNA that is unfavorable to the stress response, aberrant mRNA is also degraded by P-Bodies. It is worth noting that mRNA that is beneficial to stress response, such as the mRNA of heat stress transcription factors (HSFs), is also stored in the P-Body to prevent degradation [28].

**Figure 1:**
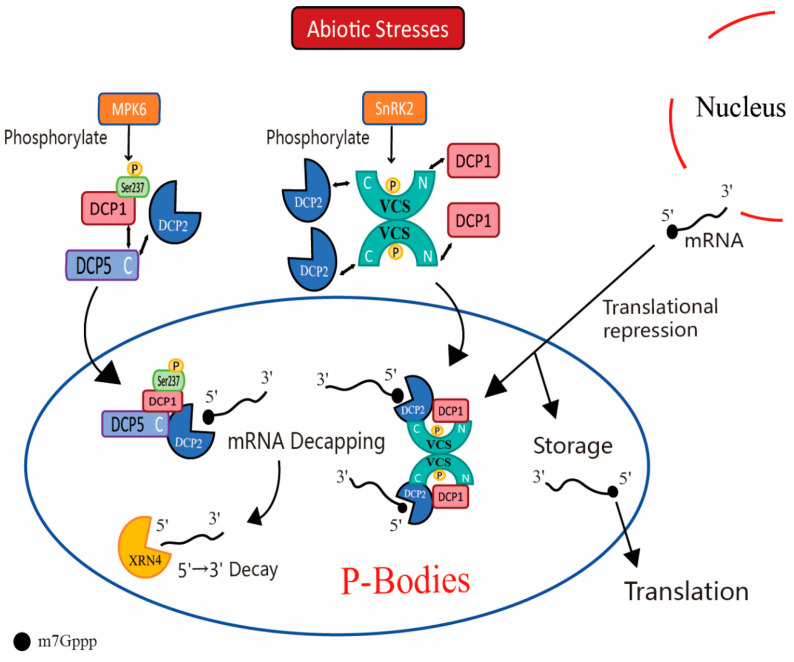
The P-Body responds to abiotic stresses in plants by regulating mRNA uncapping, degradation, translational repression, and storage at the post-transcriptional level. In plant cells, abiotic stresses including osmotic and temperature stresses activate kinase MPK6 and SnRK2 to phosphorylate DCP1 and VCS, respectively, and through this regulate P-Body assembly under stress. Phosphorylated DCP1 interacts with DCP2 and DCP5, and phosphorylated VCS interacts with DCP1 and DCP2, resulting in decapping complexes to excise the 5′-m7Gpp-protecting structure of mRNAs unfavorably resistant or aberrantly transcribed. The decapped mRNA is subsequently degraded by 5′→3′ exoribonuclease XRN4. mRNAs functionally involved in the stress response and mRNAs required for plant growth to adapt to the normal environment are selectively recruited into the P-Body for storage and protection. But whether the mRNAs are degraded or stored in the P-Body, their translation is repressed.

The proteins of the P-Body can be categorized into conserved proteins and dynamic proteins. DCP1, DCP2, DCP5, VCS, and XRN4 are conserved proteins of the P-Body and play crucial roles in the 5′→3′ degradation process of mRNA [10,22]. Abiotic stresses activate kinase MPK6 and SnRK2 to phosphorylate DCP1 and VCS, respectively, promoting DCP1, DCP2, DCP5, and VCS to form decapping complexes to excise the 5′-m7Gpp protecting structure of mRNAs unfavorably resistant or aberrantly transcribed [55,56]. And the decapped mRNA is subsequently degraded by 5′→3′ exoribonuclease XRN4 (Figure 1) [60]. It is interesting to note that under biotic stress, in plant cell pattern recognition receptors (PRR)-mediated plant immunity against pathogens, MPK6 also phosphorylates DCP1, but the 5′→3′ degradation process of mRNAs changes. Yu et al. found that the flg22, known to induce this immune response, activates the kinases MPK3 and MPK6 when entering cells. The MPK3 and MPK6 phosphorylate DCP1 when the mRNA is decapped by the decapping complex in P-Body. The phosphorylated DCP1 then dissociates from the decapping complex and associates with the nucleic acid exonuclease XRN4 [71]. The association of phosphorylated DCP1 with XRN4 may further promote the degradation of mRNA by the nucleic acid exonuclease XRN4. This may indicate that the role of MPK6 in phosphorylating DCP1 is different under abiotic and biotic stresses. Under abiotic stress, MPK6 promotes the formation of a decapping complex of DCP1 with DCP2 and DCP5 to promote mRNA decapping and the formation of the P-Body. In contrast, under biotic stress, MPK6 promotes the detachment of DCP1 from the decapping complex and its association with the nucleic acid exonuclease XRN4, which promotes the degradation of decapitated mRNA and the disassembly of the P-Body. The disassembly of the P-Body plays an important role in plant immune responses under biotic stress. The above phenomenon suggests that the assembly and disassembly of the P-Body is a result of the different functions performed by the components of P-Body, reflecting the dynamic nature of membrane-less organelles such as the P-Body. Besides mRNA degradation, the P-Body might also inhibit translation and store the mRNA under stresses. This facilitates the rapid translation of mRNAs required for plant growth to adapt to normal environments and protect mRNAs functionally involved in the stress response (Figure 1).

Currently, the dynamic protein composition of the P-Body remains unclear. This is due to the fact that the components of the P-Body are affected by factors such as cell type, growth period, and external environmental stress, showing a complex and dynamic regulatory mechanism. Under abiotic stress, the dynamic proteins of the P-Body—CCCH-zinc finger (zf_CCCH) proteins, acetylation lowers binding affinity (ALBL) proteins, and Tudor staphylococcal nuclease (TSN) protein—can dynamically traffic between the nucleus and P-Body/stress granules, thereby cooperatively regulating the expression of related stress-responsive genes at the transcriptional and post-transcriptional levels [26,27,28,57,64,72,73]. This reflects the complex and dynamic regulatory mechanisms of P-Body in response to stress.

The P-Body can also enhance stress resistance and regulate plant growth by regulating the expression of genes associated with other hormone pathways. In the ethylene signaling pathway, EIN2 directs the 1U-containing mRNA to P-Body, where it inhibits translation, thereby promoting the sensitivity of the plant responding to ethylene [66]. Therefore, exploring the functions played by the P-Body in the hormonal pathways of plants will help to further understand how the P-Body regulates plants’ growth and enhances plant resistance under abiotic stress.

## Figures and Tables

**Table 1:** Proteins containing disordered regions in P-Body.

Protein	Uniprot	Experimental Method	Species	References
DCP1	ACZ94554.1	Prediction	*D* *. melanogaster*	[34]
DCP2	AFH04447.1	Prediction	*D* *. melanogaster*	[34,35]
DCP5	OAP16255.1	predicted by D2P2	*A* *. thaliana*	[36]
Me31B	EDW88764.1	Prediction	*Drosophila*	[37]
NBDY	NP_001335058.1	1H-15N HSQC	*H* *. sapiens*	[38]
Sts5	BAA23619.1	Prediction	*S. pombe*	[39]
Puf2	NP_595389.2	Prediction	*S* *. pombe*	[40]
Puf3	NP_593141.2	Prediction	*S* *. pombe*	[40]
Puf4	CAB03616.1	Prediction	*S* *. pombe*	[40]
SMG7	OAO95115.1	Prediction	*A. thaliana*	[33]
Lsm4	QHB08189.1	Prediction	*S* *. cerevisiae*	[41]
Dhh1	CAA98734.1	Prediction	*S* *. cerevisiae*	[41]
Pop2	CAA96333.1	Prediction	*S* *. cerevisiae*	[41]
Ccr4	AAB24455.1	Prediction	*S* *. cerevisiae*	[41]
Nst1	QHB11286.1	Prediction	*S. cerevisiae*	[32]
G3BP	CAG38772.1	Prediction	*H* *. sapiens*	[42]
MEG-1	NP_510319.1	Prediction	*C. elegans*	[43]
MEG-2	NP_510318.2	Prediction	*C. elegans*	[43]
